# Minimally invasive versus open abdominoperineal resection and the risk of postoperative perineal hernia: a systematic review and meta-analysis

**DOI:** 10.1007/s10029-026-03841-1

**Published:** 2026-09-09

**Authors:** Beatriz Yukari Yokoyama, Victória Recidivi e Silva, Lucas Carvalho Lemos de Souza Aguiar, Hélder Santos Gonçalves, Lucas Monteiro Delgado, Claudia Theis, Sérgio Mazzola Poli de Figueiredo, Fernanda Bellotti Formiga, Bernardo Fontel Pompeu

**Affiliations:** 1 Department of Surgery, Heliopolis Hospital, São Paulo, SP Brazil; 2 USCS – University of São Caetano do Sul, Rua Santo Antônio, 50 - Centro, São Caetano do Sul, SP 09521-160 Brazil; 3 Federal University of Minas Gerais, Belo Horizonte, Brazil; 4 Department of Surgery, University of North Carolina, Chapel Hill, NC United States; 5 Department of Colorectal Surgery, Hospital of Santa Casa de Misericórdia, São Paulo, Brazil

**Keywords:** Abdominoperineal resection, Extralevator abdominoperineal excision, Perineal hernia, Minimally invasive surgery, Rectal cancer, Meta-analysis

## Abstract

**Background:**

The impact of minimally invasive surgery on the risk of postoperative perineal hernia after abdominoperineal resection (APR) or extralevator abdominoperineal excision (ELAPE) remains uncertain. This study compares perineal hernia rates and perioperative outcomes between minimally invasive and open approaches.

**Methods:**

PubMed, Scopus, Web of Science, and Cochrane Library were searched through June 2026. Pooled odds ratios (ORs) and mean differences (MDs) with 95% confidence intervals (CIs) were calculated using random-effects models. A Bayesian meta-analysis was additionally performed for the primary outcome.

**Results:**

Four comparative observational studies involving 763 patients were included; 249 underwent minimally invasive APR/ELAPE, and 514 underwent open APR/ELAPE. Postoperative perineal hernia was significantly more frequent following minimally invasive surgery (OR 4.13; 95% CI 2.24–7.61; *p* < 0.001). Intraoperative blood loss was significantly lower in the minimally invasive group (MD − 156.5 mL; 95% CI − 298.4 to − 14.5; *p* = 0.03), as was operative time (MD − 41.7 min; 95% CI − 60.8 to − 22.5; *p* < 0.01). No significant differences were observed in hospital stay (MD − 2.5 days; 95% CI − 5.4 to 0.4; *p* = 0.09) or 30-day readmission rates (OR 1.41; 95% CI 0.82–2.42; *p* = 0.209). Bayesian analysis yielded a posterior mean OR of 4.04 (95% CrI 1.96–8.36), corresponding to a 99.9% posterior probability that minimally invasive surgery increases the risk of postoperative perineal hernia.

**Conclusion:**

Minimally invasive APR/ELAPE was associated with an increased risk of postoperative perineal hernia compared with the open approach. Strategies to reduce this complication while preserving the benefits of minimally invasive surgery warrant further investigation.

**Supplementary Information:**

The online version contains supplementary material available at https://doi.org/10.1007/s10029-026-03841-1.

## Introduction

 Perineal hernia is an uncommon but clinically relevant complication after abdominoperineal resection (APR) or extralevator abdominoperineal excision (ELAPE). It is characterized by the protrusion of intra-abdominal contents through a defect in the pelvic floor [1, 2]. Reported incidence varies widely, ranging from less than 1% in older surgical series to more than 20% in selected cohorts, reflecting differences in surgical technique, follow-up duration, diagnostic methods, and whether asymptomatic radiological hernias are included [1–4]. Although some patients remain asymptomatic, perineal hernia may cause perineal bulging, discomfort, pain, urinary dysfunction, bowel obstruction, skin complications, and impaired quality of life [1, 2, 4–6].

Several factors have been implicated in the development of postoperative perineal hernia, including female sex, previous hysterectomy, coccygectomy, neoadjuvant radiotherapy, perineal wound infection, extensive pelvic floor resection, and failure to close or reinforce the pelvic peritoneum [1–4]. The increasing use of ELAPE, which creates a wider pelvic floor defect, and the widespread adoption of minimally invasive surgery have renewed interest in this complication [3, 4, 6]. A biologically plausible hypothesis is that minimally invasive approaches may result in fewer intra-abdominal adhesions, allowing small bowel loops to descend more easily into the pelvis after APR or ELAPE [3, 4, 6].

Despite this hypothesis, the relationship between minimally invasive surgery and postoperative perineal hernia remains incompletely defined. Existing studies are limited by retrospective design, heterogeneous populations, variable closure or reconstruction techniques, and inconsistent follow-up [1–4]. Given the potential trade-off between the perioperative benefits of minimally invasive surgery and the possible increased risk of perineal hernia, a comprehensive synthesis of the comparative literature is warranted. Therefore, this systematic review and meta-analysis aimed to compare postoperative perineal hernia and perioperative outcomes between minimally invasive and open APR/ELAPE.

## Methods

### Protocol and registration

The present systematic review and meta-analysis were undertaken in accordance with the Preferred Reporting Items for Systematic Reviews and Meta-Analyses (PRISMA) recommendations, with the corresponding checklist available in Supplementary Table S1 [7]. To promote transparency and methodological consistency while minimizing reporting bias, the protocol was prospectively registered in the International Prospective Register of Systematic Reviews (PROSPERO; CRD420261422529) prior to conducting the review [8].

### Systematic search process

A comprehensive electronic search was conducted in PubMed, Scopus, the Cochrane Central Register of Controlled Trials, and Web of Science from database inception through June 2026. Keywords and subject headings related to abdominoperineal resection, extralevator abdominoperineal excision, perineal hernia, and minimally invasive versus open surgery were combined with Boolean operators to maximize study retrieval. The search strategy applied across PubMed was as follows: (“abdominoperineal resection” OR “abdominoperineal excision” OR APR OR APE OR ELAPE OR “extralevator abdominoperineal excision” OR “extralevator abdominoperineal resection”) AND (“perineal hernia” OR “postoperative perineal hernia” OR “pelvic floor hernia”) AND (laparoscopic OR laparoscopy OR robotic OR “robot-assisted” OR “minimally invasive” OR open). An equivalent strategy, adapted to database-specific indexing terms and syntax, was applied to Scopus, Cochrane, and Web of Science.

Records identified through database searching were imported into Zotero (Corporation for Digital Scholarship, Vienna, VA, USA) for duplicate removal and subsequently uploaded to Rayyan software (Qatar Computing Research Institute, Qatar Foundation, Doha, Qatar). Two reviewers (B.Y.Y. and V.R.S.) independently performed title and abstract screening, followed by full-text review of potentially eligible studies. Disagreements were resolved by consensus or, when required, by consultation with a third reviewer (B.F.P.).

### Study selection criteria

The review question and eligibility criteria were formulated according to the PICOS framework. Studies were considered eligible if they fulfilled all of the following criteria: included adult patients undergoing abdominoperineal resection (APR) or extralevator abdominoperineal excision (ELAPE); compared minimally invasive approaches (laparoscopic or robotic) with open surgery; and reported postoperative perineal hernia, pelvic floor hernia, or radiologically diagnosed perineal hernia as an outcome. Both comparative observational studies (prospective or retrospective cohort studies) and randomized controlled trials were considered for inclusion when available. No language restrictions were applied during the literature search or study selection. Studies were excluded if they: reported hernia outcomes without specifying a perineal location; focused exclusively on the management or repair of perineal hernias rather than their incidence; lacked an open surgical comparison group; evaluated preventive strategies alone (such as mesh reinforcement or pelvic floor reconstruction) without stratification according to surgical approach; were reviews, editorials, letters, case reports, or technical descriptions without extractable comparative data; or included mixed hernia populations in which perineal hernia outcomes could not be distinguished from incisional, parastomal, or internal hernias.

### Data extraction and endpoints

Data extraction was independently performed by two reviewers (B.Y.Y. and V.R.S.) using a standardized electronic form. Disagreements were resolved by consensus or consultation with a third reviewer (B.F.P.). Extracted data included study characteristics (author, publication year, country, study period, design, and sample size), baseline patient characteristics (sex, age, body mass index, diabetes, smoking status, and follow-up), tumor-related variables (location, neoadjuvant therapy, and AJCC stage), perineal closure or reconstruction techniques, and combined pelvic resection rates when available. Postoperative perineal hernia, diagnosed clinically or confirmed radiologically following APR or ELAPE, was defined as the primary outcome. Secondary outcomes comprised operative time, intraoperative blood loss, length of hospital stay, and 30-day readmission.

### Quality assessment

The methodological quality of the included studies was independently evaluated by two reviewers using the Risk Of Bias In Non-randomized Studies of Interventions (ROBINS-I) tool [9]. The assessment encompassed seven domains: confounding, participant selection, intervention classification, deviations from intended interventions, missing data, outcome measurement, and selective reporting. Each study was classified as having low, moderate, serious, or critical risk of bias within each domain and overall, in accordance with ROBINS-I recommendations. The results were graphically summarized using the RobVis tool. Any disagreements were resolved through discussion and consensus, with involvement of a third reviewer when required.

The certainty of evidence was assessed using the GRADE (Grading of Recommendations Assessment, Development and Evaluation) methodology and classified as high, moderate, low, or very low [10]. The corresponding evaluations are presented in the Summary of Findings table.

### Statistical analyses

Dichotomous outcomes were synthesized using odds ratios (ORs) with 95% confidence intervals (CIs), whereas continuous outcomes were pooled as mean differences (MDs) with 95% Cis. Given the anticipated clinical and methodological variability across studies, all analyses were conducted using random-effects models [11, 12]. Statistical heterogeneity was quantified using the I² statistic, with higher values indicating greater between-study inconsistency. For outcomes with at least moderate heterogeneity (I² ≥ 25%) [13], sensitivity analyses were performed using Baujat plots to identify studies that disproportionately contributed to heterogeneity and overall effect estimates, followed by leave-one-out analyses to assess the influence of individual studies on the pooled results [14].

A Bayesian random-effects meta-analysis was additionally conducted for the primary outcome (perineal hernia) using weakly informative priors [µ ~ Normal(0, 2²); τ ~ Half-Normal(0.5)]. Sensitivity analyses employing alternative skeptical [µ ~ Normal(0, 0.8²); τ ~ Half-Normal(0.25)] and diffuse [µ ~ Normal(0, 4²); τ ~ Half-Normal(1.0)] priors were performed to assess the robustness of the findings (Supplementary Table 2). All analyses were conducted using R software, and a two-sided p-value < 0.05 was considered statistically significant for the frequentist analyses [15–18].

## Results

### Study inclusion and baseline characteristics

**Fig. 1:**
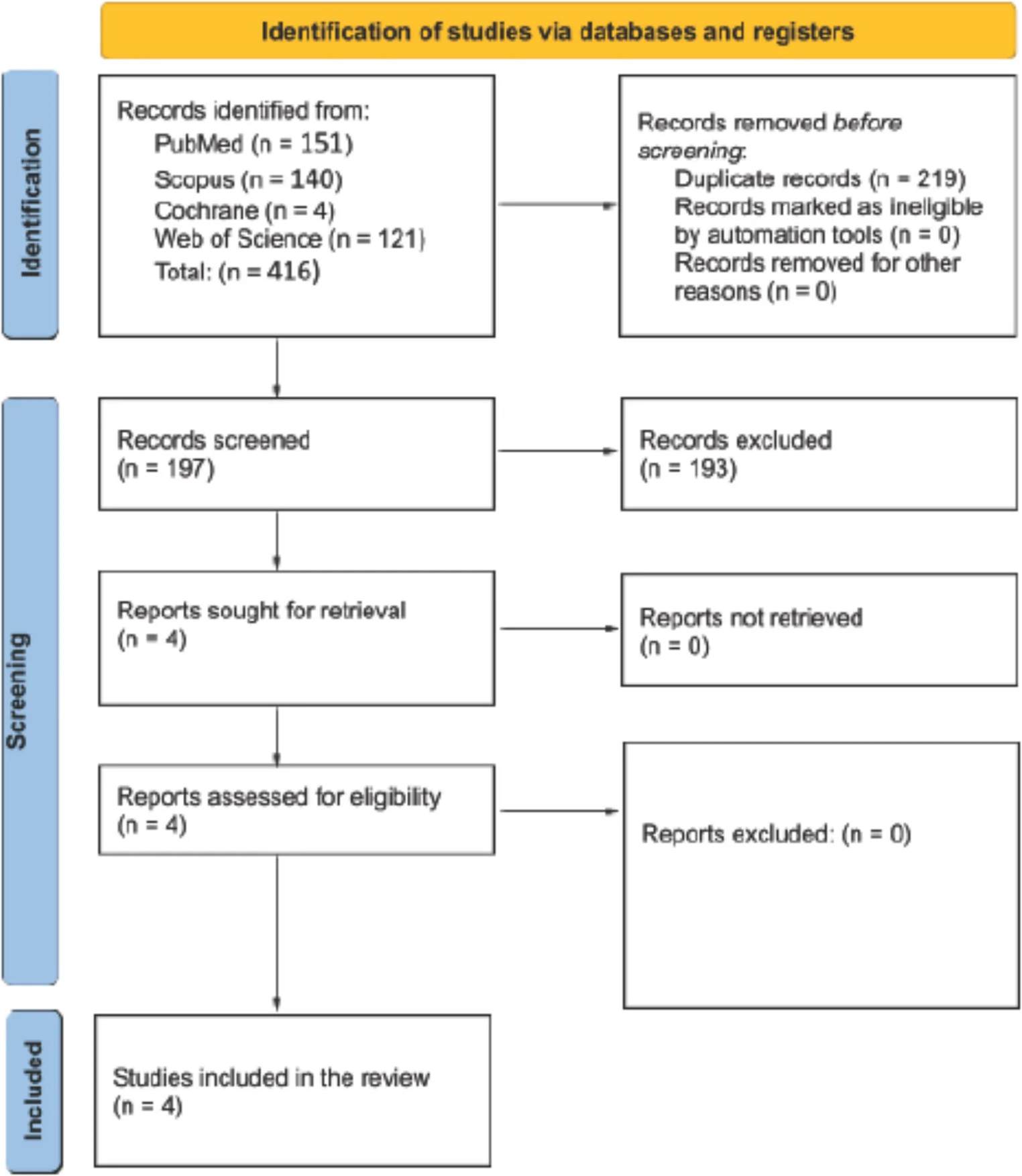
PRISMA flow diagram of study screening and selection

The study selection process is illustrated in Fig. 1. The literature search identified 416 records across PubMed, Scopus, the Cochrane Central Register of Controlled Trials, and Web of Science. After removal of 219 duplicate records, 197 studies underwent title and abstract screening, of which 193 were excluded. Ultimately, four retrospective comparative studies were included in the qualitative and quantitative synthesis [19–22]. A total of 763 patients were included, of whom 249 (32.6%) underwent minimally invasive APR/ELAPE, and 514 (67.4%) underwent open APR/ELAPE. The included studies were published between 2015 and 2022 and originated from Canada, the United Kingdom, China, and the United States [19–22]. All studies were retrospective comparative cohorts. Three studies exclusively evaluated patients undergoing ELAPE [20–22], whereas the remaining study primarily included conventional APR, with selected ELAPE procedures performed for locally advanced tumors without reporting procedure-specific outcomes [19]. Across the pooled cohort, the proportion of male patients was slightly higher in the open group than in the minimally invasive group (64.6% vs. 57.6%). Mean age was slightly higher among patients undergoing minimally invasive surgery (61.75 ± 13.62 years) compared with open surgery (59.45 ± 13.73 years), whereas BMI was comparable between groups (24.50 ± 5.23 vs. 25.20 ± 4.93 kg/m²) [19–22]. Diabetes was more frequently reported in the open group (14.2% vs. 6.9%), while smoking was slightly more common in the minimally invasive group (28.7% vs. 24.8%). Mean follow-up duration was similar between groups (32.76 ± 34.18 vs. 33.56 ± 24.37 months) [19–22]. Most patients underwent surgery for rectal cancer (94.8% in the minimally invasive group and 91.7% in the open group). Anal cancer accounted for a small proportion of cases (1.7% vs. 2.1%), while non-malignant indications represented 3.5% and 6.2% of patients, respectively [19–22]. Neoadjuvant radiotherapy and chemotherapy were administered more frequently in the open group (40.2% and 38.1%, respectively) than in the minimally invasive group (32.8% and 27.6%, respectively). Conversely, chemoradiotherapy was more frequently reported in the minimally invasive cohort (34.6% vs. 5.7%), largely driven by a single study [19–22]. AJCC stage distribution was broadly comparable between groups. Stage III disease was the most common stage in both the minimally invasive and open cohorts (36.2% vs. 35.0%), followed by stage II disease (31.6% vs. 24.2%) [19–22]. Stage 0 was present in 27.1% and 31.9% of patients, stage I in 11.5% and 11.8%, and stage IV in 8.6% and 9.4% of patients in the minimally invasive and open groups, respectively [19–22]. Linear or primary suture was the most frequently used method of perineal closure in both groups (82.9% vs. 83.8%). Purse-string closure was more commonly performed in the minimally invasive cohort (11.4% vs. 2.6%) [19–22]. Flap-based reconstruction was uncommon overall, including TRAM flaps (1.4% vs. 7.3%), gracilis flaps (1.4% vs. 3.7%), and other flap techniques (1.4% vs. 2.1%). Combined pelvic resection was more frequently reported in the open group than in the minimally invasive group (14.8% vs. 9.2%) [19–22]. Pooled baseline demographic, oncologic, and operative characteristics are summarized in Tables 1 and 2, and Supplementary Table S3.

**Table 1:** Study and patient characteristics of included cohorts

Study	Country	MIS/Open (*n*)	Design	Study Period	Male Sex, *n* (%) MIS/Open	Age, years MIS/Open	BMI, kg/m² MIS/Open	Diabetes, *n* (%) MIS/Open	Smoking, *n* (%) MIS/Open	Follow-up, months MIS/Open
Black [19]	Canada	70 / 191	R-Obs	2007–2018	45 (64.3) / 125 (65.4)	62.61 ± 14.33 / 57.57 ± 13.78	26.45 ± 6.54 / 26.23 ± 5.57	8 (11.6) / 32 (16.9)	8 (11.6) / 31 (16.5)	20 ± 17.8 / 29.3 ± 23.1
Shen [20]	China	104 / 140	R-Obs	2014–2019	59 (56.7) / 90 (64.3)	62 (26–91) / 59.5 (22–91)	22.9 (13.9–30.1) / 23.3 (15.9–31.3)	4 (3.8) / 15 (10.7)	42 (40.4) / 51 (36.4)	47.5 (9–77) / 54.5 (12–77)
Sayers [21]	UK	20 / 34	R-Obs	2008–2012	40 (74.1)*	69.5 (31–90)*	NR	6 (11.1)*	NR	38 (9–61)*
Kent [22]	USA	55 / 149	R-Obs	2010–2020	28 (50.9) / 95 (63.7)	63.48 ± 13.9 / 63.23 ± 13.5	25.9 ± 5.1 / 25.53 ± 5.12	NR	NR	25.4 ± 60.1 / 24.05 ± 27.15

Abbreviations: *MIS* minimally invasive surgery, *BMI* body mass index, *R-Obs* retrospective observational study, *SD* standard deviation, *NR* not reported. * Arm-level data not reported; value shown is for the overall study cohort

**Table 2:** Tumor characteristics and treatment patterns of included studies

Study	Tumor location, *n* (%) MIS/Open	Neoadjuvant therapy, *n* (%) MIS/Open	AJCC Stage, *n* (%) MIS/Open	Perineal reconstruction/Closure, *n* (%) MIS/Open	Combined pelvic resection, *n* (%) MIS/Open
Black [19]	Anal: 4 (5.7)/10 (5.2) Rectal: 58 (82.9)/151 (79.0) NC: 8 (11.4)/30 (15.7)	RT: 52 (75.4)/133 (71.1) CT: 45 (62.2)/119 (63.3)	0: 19 (27.1)/61 (31.9) I: 18 (25.7)/26 (13.6) IIA: 16 (22.9)/27 (14.1) IIB: 1 (1.4)/11 (5.8) IIC: 1 (1.4)/3 (1.6) IIIA: 5 (7.1)/11 (5.8) IIIB: 4 (5.7)/37 (19.4) IIIC: 0 (0.0)/1 (0.5) IV: 4 (5.7)/10 (5.2)	LS: 58 (82.9)/160 (83.8) PS: 8 (11.4)/5 (2.6) TRAM: 1 (1.4)/14 (7.3) GF: 1 (1.4)/7 (3.7) OF: 1 (1.4)/4 (2.1)	7 (10.0)/41 (21.5)
Shen [20]	Rectal: 104/140	RT: 5 (4.8)/0 (0) CT: 3 (2.9)/7 (5.0) CRT: 36 (34.6)/8 (5.7)	I: 2 (1.9)/13 (9.3) II: 37 (35.6)/39 (27.9) III: 54 (51.9)/67 (47.9) IV: 11 (10.6)/21 (15.0)	NR	9 (8.7)/6 (4.3)
Sayers [21]	NR	CRT: 52 (96.3)*	NR	MMF: 6 (11.1)* BM: 2 (3.7)* PC: 46 (85.2)*	NR
Kent [22]	Rectal: 55/149	NR	NR	RAMF: 0 (0.0)/69 (46.3)	5 (9.1)/24 (16.1)

Abbreviations: *NC* no cancer, *RT* radiotherapy, *CT* chemotherapy, *CRT* chemoradiotherapy, *PC* primary closure, *LS* linear suture, *PS* purse-string suture, *TRAM* transverse rectus abdominis myocutaneous flap, *GF* gracilis flap, *OF* other flap, *MMF* myocutaneous flap, *BM* biological mesh, *RAMF* rectus abdominis musculocutaneous flap, *AJCC* American Joint Committee on Cancer, *NR* not reported; * arm-level data not reported

### Pooled analyses of the included studies

#### Frequentist outcomes

**Fig. 2:**
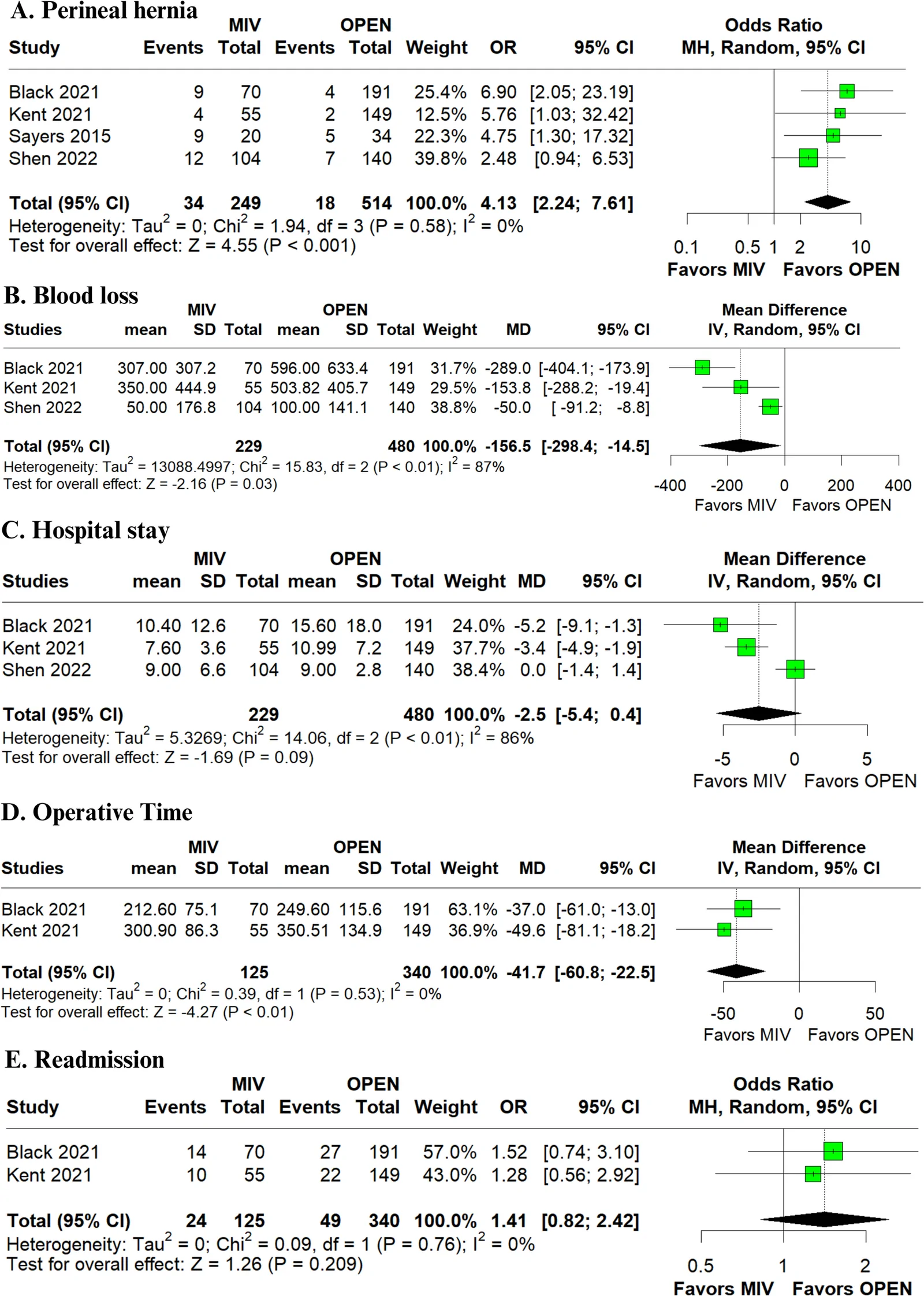
Forest plots comparing minimally invasive and open abdominoperineal resection/extralevator abdominoperineal excision for (**A**) postoperative perineal hernia, (**B**) intraoperative blood loss, (**C**) length of hospital stay, (**D**) operative time, and (**E**) 30-day readmission. MIV = minimally invasive (consistent with MIS in Tables 1 and 2); OPEN = open approach

In the pooled cohort, postoperative perineal hernia occurred in 34 of 249 patients (13.7%) undergoing minimally invasive surgery and in 18 of 514 patients (3.5%) undergoing open surgery. Accordingly, minimally invasive surgery was associated with a significantly higher risk of postoperative perineal hernia (OR 4.13, 95% CI 2.24–7.61; *p* < 0.001; I² = 0%; Fig. 2A), with no evidence of statistical heterogeneity. Similarly, intraoperative blood loss was significantly lower in the minimally invasive group (MD − 156.5 mL, 95% CI − 298.4 to − 14.5; *p* = 0.03; I² = 87%; Fig. 2B), although substantial between-study heterogeneity was observed. Operative time was also significantly lower in the minimally invasive group (MD − 41.7 min, 95% CI − 60.8 to − 22.5; *p* < 0.01; I² = 0%; Fig. 2D), with no heterogeneity detected. No significant differences were observed in length of hospital stay (MD − 2.5 days, 95% CI − 5.4 to 0.4; *p* = 0.09; I² = 86%; Fig. 2C) or 30-day readmission rates (OR 1.41, 95% CI 0.82–2.42; *p* = 0.209; I² = 0%; Fig. 2E).

#### Frequentist sensitivity analyses

Sensitivity analyses were performed for outcomes demonstrating substantial heterogeneity, namely intraoperative blood loss and hospital stay. For hospital stay, the Baujat plot identified Shen 2022 as the principal contributor to between-study heterogeneity. Exclusion of this study completely eliminated heterogeneity (I² from 86% to 0%) and resulted in a statistically significant reduction in hospital stay, favoring the minimally invasive approach (MD − 3.62 days, 95% CI − 5.01 to − 2.23; Supplementary Figures S1–S2). For intraoperative blood loss, Black 2021 was identified as the main contributor to heterogeneity. Exclusion of this study resulted in loss of statistical significance (MD − 81.4 mL, 95% CI − 174.8 to 12.1, Supplementary Figures S3–S4), while heterogeneity decreased only modestly (I² from 87% to 52%).

#### Bayesian outcomes

**Fig. 3:**
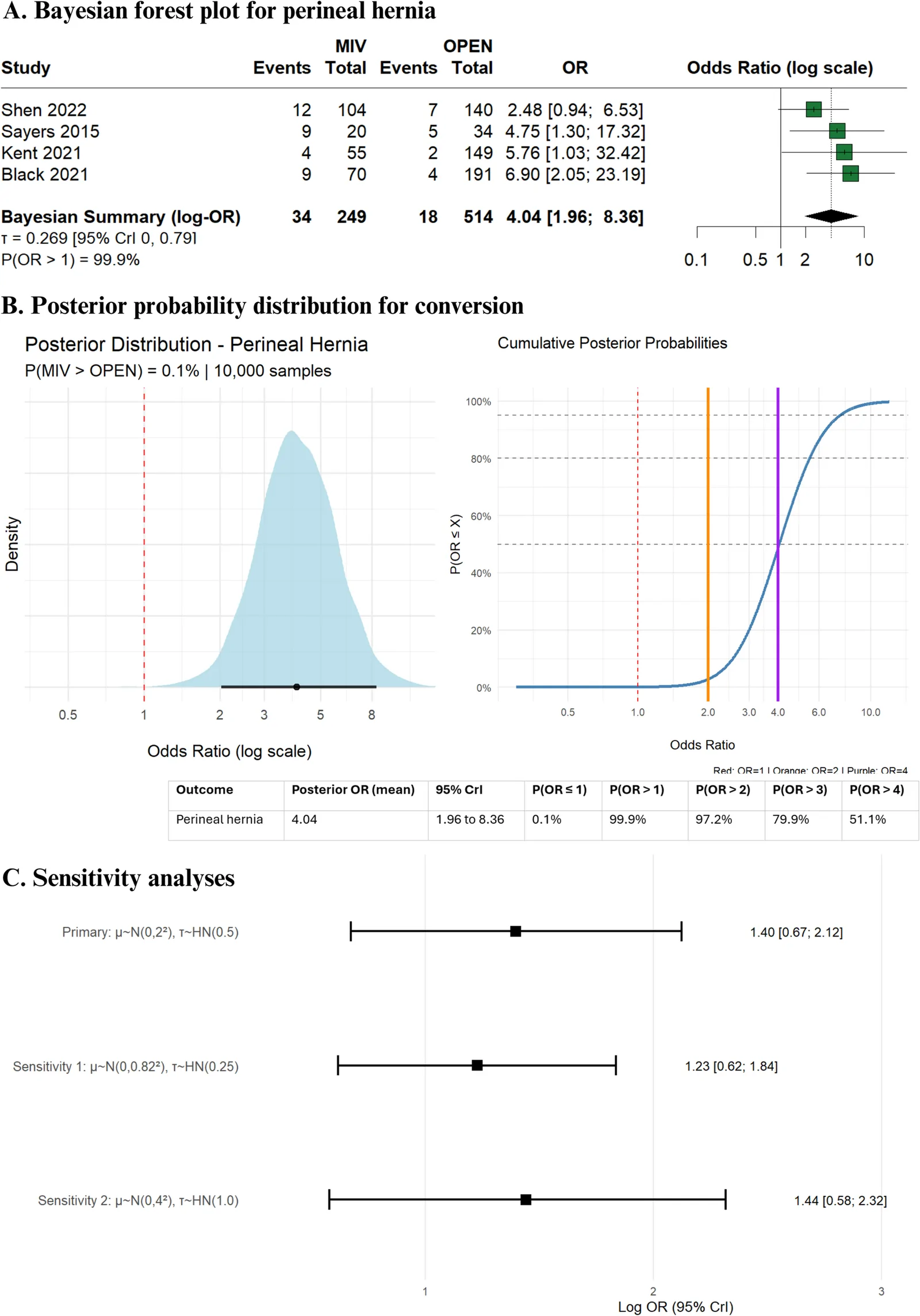
Bayesian analysis of postoperative perineal hernia showing (**A**) Bayesian Forest plot, (**B**) posterior probability distribution and cumulative posterior probabilities, and (**C**) sensitivity analyses using alternative prior distributions. MIV = minimally invasive (consistent with MIS in Tables 1 and 2)

The Bayesian analysis yielded a posterior mean OR of 4.04 (95% CrI 1.96–8.36; Fig. 3A), corresponding to a 99.9% posterior probability that minimally invasive APR/ELAPE increases the risk of postoperative perineal hernia compared with open surgery. The probability that minimally invasive surgery was associated with at least a twofold increase in perineal hernia risk was 97.2%, whereas the probabilities of OR > 3 and OR > 4 were 79.9% and 51.1%, respectively (Fig. 3B). Bayesian sensitivity analyses demonstrated consistent results across alternative prior distributions (Fig. 3C), with all models indicating a greater than 99% probability that minimally invasive surgery increases the risk of postoperative perineal hernia, supporting the robustness of the primary findings. All analyses were summarized in Supplementary table S4.

### Quality assessment and certainty

**Fig. 4:**
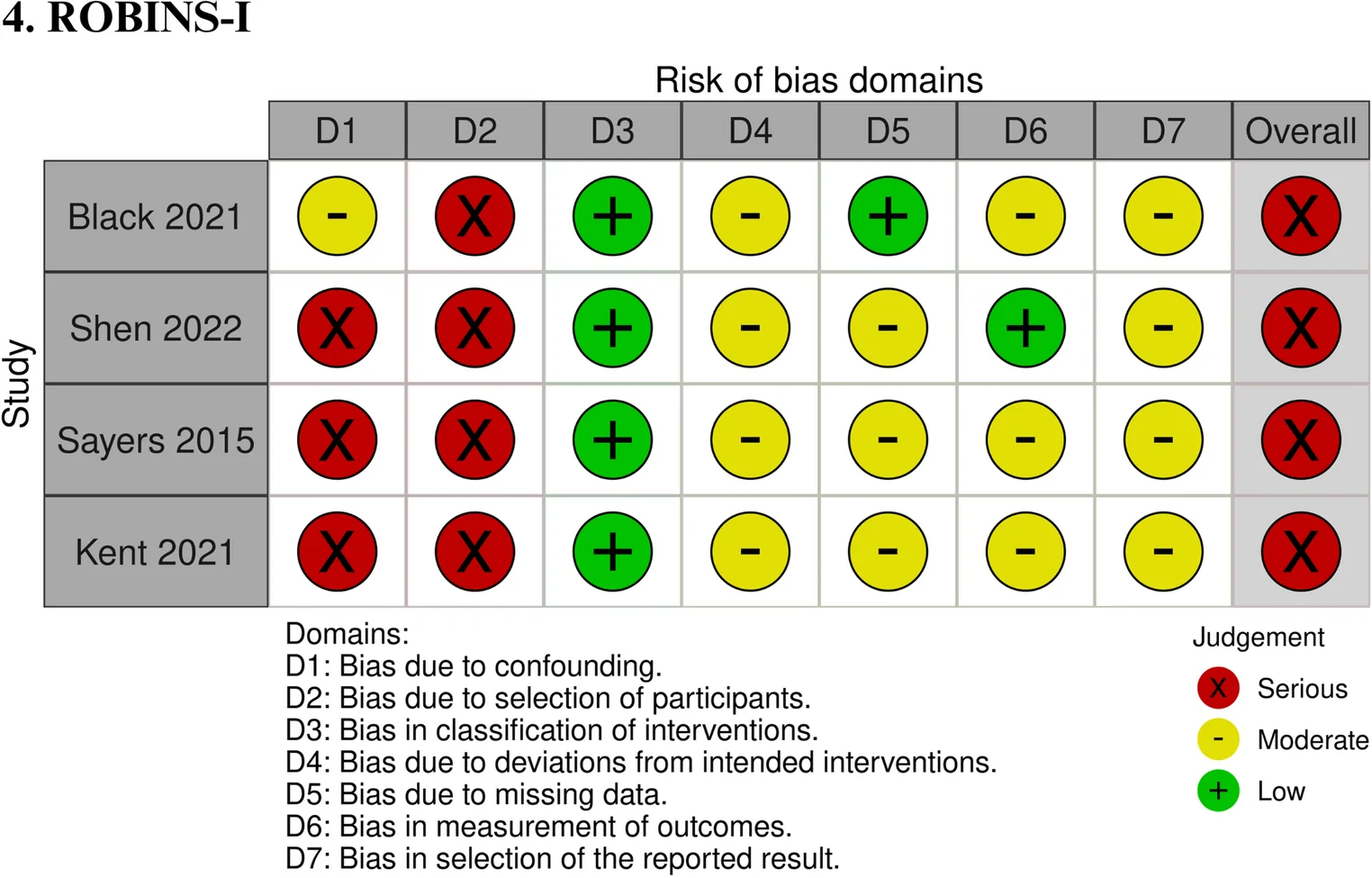
Risk-of-bias assessment of the included studies using the ROBINS-I tool

The risk-of-bias assessment is summarized in Fig. 4 and Supplementary Table S5. According to the ROBINS-I tool, all four included studies were judged to have a serious overall risk of bias. The main sources of bias were confounding (D1) and participant selection (D2), which received serious ratings in most studies. Three studies did not employ multivariable adjustment or propensity score methods and were therefore judged at serious risk of bias due to confounding, whereas Black 2021 achieved a moderate rating through multivariable adjustment. Selection bias related to the retrospective observational design resulted in a serious rating for D2 across all studies. In contrast, all studies were judged to be at low risk for intervention classification (D3). The remaining domains (D4–D7) were predominantly rated as moderate risk, with isolated low-risk assessments for missing data (D5) and outcome measurement (D6).

According to the GRADE framework, the certainty of evidence was rated as very low for all evaluated outcomes, including perineal hernia, operative time, hospital stay, intraoperative blood loss, and readmissions. The certainty ratings were primarily downgraded because all included studies were observational and judged to be at serious risk of bias. Additional downgrading occurred for inconsistency in outcomes with substantial heterogeneity and for imprecision when confidence intervals were wide or the number of contributing studies was limited. The complete Summary of Findings and GRADE assessment is presented in Supplementary Table S6.

## Discussion

In this systematic review and meta-analysis, including four comparative observational studies and 763 patients undergoing APR or ELAPE, minimally invasive surgery was associated with a significantly higher risk of postoperative perineal hernia compared with the open approach. This finding was remarkably consistent across all included studies, with no statistical heterogeneity, and was further supported by the Bayesian analysis, which demonstrated a 99.9% posterior probability that minimally invasive surgery increases the risk of perineal hernia. Importantly, these findings remained stable across alternative prior assumptions. In contrast, minimally invasive surgery was associated with lower intraoperative blood loss and shorter operative time, whereas no significant differences were observed in hospital stay or 30-day readmission rates between approaches.

The increased risk of perineal hernia after minimally invasive APR/ELAPE is biologically plausible and has been suggested by previous studies [19–22]. Laparoscopic and robotic approaches are associated with reduced postoperative intra-abdominal adhesion formation compared with open surgery, which may facilitate descent of abdominal viscera through the residual pelvic floor defect after rectal resection [1, 4, 6]. This mechanism may be further accentuated after ELAPE, when wider levator muscle resection creates a larger pelvic floor defect [1, 4, 6]. These concepts have previously been discussed by Mjoli et al. and Goedhart-de Haan et al. and are supported by more recent comparative studies [1, 6]. Black et al. directly compared laparoscopic and open APR and reported perineal hernia rates of 12.9% and 2.1%, respectively, identifying laparoscopic surgery as an independent risk factor on multivariable analysis [19]. Likewise, Sayers et al. reported an overall perineal hernia rate of 26% after ELAPE, with a disproportionately high incidence following laparoscopic surgery, while Shen et al. demonstrated higher hernia rates after laparoscopic than open ELAPE (11.5% vs. 5.0%) [20, 21]. More recently, Kasai et al. reported a 37.3% radiological incidence of perineal hernia one year after robotic APR and identified preoperative chemoradiotherapy and the absence of pelvic reinforcement as independent risk factors [4]. Collectively, these findings are consistent with the results of the present meta-analysis and reinforce the concept that postoperative perineal hernia is a multifactorial complication influenced by both surgical approach and pelvic reconstruction.

It’s noteworthy that, rather than discouraging the use of minimally invasive APR or ELAPE, our findings highlight the need to develop effective strategies to reduce postoperative perineal hernia while preserving the well-established perioperative benefits of minimally invasive surgery. Several preventive approaches have been proposed, including pelvic peritoneal closure, prophylactic mesh reinforcement, and flap-based reconstruction [2, 5, 23]. Shen et al. reported lower perineal hernia rates when pelvic peritoneal closure was performed after laparoscopic ELAPE, whereas Kasai et al. identified the absence of pelvic reinforcement as an independent risk factor following robotic APR [4, 20]. Likewise, recent systematic reviews and series by Li et al., Yuan et al., Balla et al., and Maspero et al. have highlighted the growing interest in mesh reinforcement and reconstructive techniques for the prevention and treatment of perineal hernia [2, 3, 5, 23]. Although these strategies appear promising, the available evidence remains limited and heterogeneous, precluding definitive recommendations regarding their routine use.

The secondary outcomes were less consistent than the primary endpoint. Although minimally invasive surgery was associated with lower intraoperative blood loss and shorter operative time, blood loss showed substantial between-study heterogeneity, and the association was no longer statistically significant after exclusion of the largest study [19–22]. Similarly, hospital stay did not differ significantly in the primary analysis, but the sensitivity analysis identified Shen et al. as the principal source of heterogeneity [20]. Excluding this study eliminated heterogeneity and favored minimally invasive surgery. These findings likely reflect differences in perioperative care pathways, enhanced recovery protocols, discharge criteria, patient selection, and pelvic reconstruction techniques across studies. In contrast, readmission rates were similar between groups, suggesting that early postoperative recovery was broadly comparable despite the higher incidence of postoperative perineal hernia [19–22].

Our study represents the first comparative systematic review and meta-analysis evaluating minimally invasive versus open APR/ELAPE for postoperative perineal hernia. Heterogeneity was thoroughly explored using Baujat plots and leave-one-out analyses, allowing identification of the principal sources of between-study variability. Furthermore, the agreement between the frequentist and Bayesian analyses strengthens the credibility of the primary findings. The Bayesian model demonstrated a > 99% posterior probability that minimally invasive surgery increases postoperative perineal hernia, supporting the robustness of the findings despite the limited number of available studies.

This study has several limitations. First, all included studies were retrospective observational cohorts, limiting causal inference. Second, only four comparative studies were available, and some secondary outcomes were based on only two studies. Third, important clinical differences between cohorts, including the proportion of ELAPE procedures, methods of pelvic reconstruction, peritoneal closure, neoadjuvant treatment, and methods used to diagnose perineal hernia, may have contributed to between-study variability. Furthermore, although three included studies exclusively evaluated ELAPE, the remaining study included both conventional APR and selected ELAPE procedures without reporting procedure-specific outcomes. Consequently, separate analyses according to surgical procedure (APR versus ELAPE) were not feasible. In addition, the retrospective design of the included studies makes residual selection bias and confounding unavoidable. Surgical approach may have been influenced by disease extent, patient anatomy, surgeon preference and expertise, or the need for combined pelvic resection, while differences in radiological surveillance for perineal hernia and the higher proportion of patients receiving neoadjuvant chemoradiotherapy in the minimally invasive cohort, largely driven by a single study, may also have contributed to the observed between-study variability. Finally, although the primary outcome was remarkably consistent, the certainty of evidence remains limited, and additional prospective comparative studies are required to confirm these findings.

## Conclusion

In this meta-analysis of four comparative observational studies including 763 patients undergoing APR or ELAPE, minimally invasive surgery was associated with a significantly increased risk of postoperative perineal hernia compared with the open approach. Although minimally invasive surgery was associated with lower intraoperative blood loss and shorter operative time, no significant differences were observed in hospital stay or 30-day readmission rates. These findings highlight the importance of developing strategies to reduce perineal hernia risk while preserving the perioperative advantages of minimally invasive surgery. Further prospective studies are warranted to confirm these findings and evaluate preventive approaches for this complication.

## Supplementary Information

Below is the link to the electronic supplementary material.

Supplementary Material 1
