# Supplementary material for: Minimally invasive versus open abdominoperineal resection and the risk of postoperative perineal hernia: a systematic review and meta-analysis
Source: Hernia. 2026 Sep 9;30(1):345. doi: 10.1007/s10029-026-03841-1 (PMC13558427; doi:10.1007/s10029-026-03841-1)
Supplement: Supplementary file 1 [file 10029_2026_3841_MOESM1_ESM.docx]

**SUPPLEMENTARY MATERIAL**

**TITLE:** Minimally Invasive Versus Open Abdominoperineal Resection and the Risk of Postoperative Perineal Hernia: A Systematic Review and Meta-analysis

**SUPPLEMENTAL TABLES AND FIGURES LEGENDS:**

**Supplementary Table S1.** Preferred Reporting Items for Systematic Reviews and Meta-Analysis (PRISMA) checklist.
**Supplementary Table S2.** Study Selection and Exclusion Justifications
**Supplementary Table S3** Baseline demographic and perioperative characteristics of included patients, by suturing technique (continuous vs interrupted primary closure).

**Supplementary Table S4.** Detailed justification for risk of bias assessment according to the ROBINS-I tool.

**Supplementary Table S5.** Summary of findings and certainty of evidence assessment according to the GRADE approach.

**Supplementary Figure S1.** Baujat plot for hospital stay.

**Supplementary Figure S2.** Leave-one-out sensitivity analysis for hospital stay.

**Supplementary Figure S3.** Baujat plot for r Intraoperative blood loss .

**Supplementary Figure S4.** Leave-one-out sensitivity analysis for r Intraoperative blood loss .

**Supplementary Table S1.** Preferred Reporting Items for Systematic Reviews and Meta-Analysis (PRISMA) checklist.

| **Section and Topic** | **Item** | | **Checklist item** | **Location where item is reported** |
| --- | --- | --- | --- | --- |
| **TITLE** | | | |  |
| Title | 1 | Identify the report as a systematic review. | | Page 1 |
| **ABSTRACT** | | | |  |
| Abstract | 2 | See the PRISMA 2020 for Abstracts checklist. | | - |
| **INTRODUCTION** | | | |  |
| Rationale | 3 | Describe the rationale for the review in the context of existing knowledge. | | Pages 4-5 |
| Objectives | 4 | Provide an explicit statement of the objective(s) or question(s) the review addresses. | | Pages 4-5 |
| **METHODS** | | | |  |
| Eligibility criteria | 5 | Specify the inclusion and exclusion criteria for the review and how studies were grouped for the syntheses. | | Page 6 |
| Information sources | 6 | Specify all databases, registers, websites, organisations, reference lists and other sources searched or consulted to identify studies. Specify the date when each source was last searched or consulted. | | Pages 5-6 |
| Search strategy | 7 | Present the full search strategies for all databases, registers and websites, including any filters and limits used. | | Pages 5-6 |
| Selection process | 8 | Specify the methods used to decide whether a study met the inclusion criteria of the review, including how many reviewers screened each record and each report retrieved, whether they worked independently, and if applicable, details of automation tools used in the process. | | Pages 6-7 |
| Data collection process | 9 | Specify the methods used to collect data from reports, including how many reviewers collected data from each report, whether they worked independently, any processes for obtaining or confirming data from study investigators, and if applicable, details of automation tools used in the process. | | Pages 6-7 |
| Data items | 10a | List and define all outcomes for which data were sought. Specify whether all results that were compatible with each outcome domain in each study were sought (e.g. for all measures, time points, analyses), and if not, the methods used to decide which results to collect. | | Pages 6-7 |
|  | 10b | List and define all other variables for which data were sought (e.g. participant and intervention characteristics, funding sources). Describe any assumptions made about any missing or unclear information. | | Pages 6-7 |
| Study risk of bias assessment | 11 | Specify the methods used to assess risk of bias in the included studies, including details of the tool(s) used, how many reviewers assessed each study and whether they worked independently, and if applicable, details of automation tools used in the process. | | Page 7 |
| Effect measures | 12 | Specify for each outcome the effect measure(s) (e.g. risk ratio, mean difference) used in the synthesis or presentation of results. | | Pages 7-8 |
| Synthesis methods | 13a | Describe the processes used to decide which studies were eligible for each synthesis (e.g. tabulating the study intervention characteristics and comparing against the planned groups for each synthesis (item #5)). | | Page 6 |
|  | 13b | Describe any methods required to prepare the data for presentation or synthesis, such as handling of missing summary statistics, or data conversions. | | Pages 5 |
|  | 13c | Describe any methods used to tabulate or visually display results of individual studies and syntheses. | | Pages 6-8 |
|  | 13d | Describe any methods used to synthesize results and provide a rationale for the choice(s). If meta-analysis was performed, describe the model(s), method(s) to identify the presence and extent of statistical heterogeneity, and software package(s) used. | | Pages 6-8 |
|  | 13e | Describe any methods used to explore possible causes of heterogeneity among study results (e.g. subgroup analysis, meta-regression). | | Pages 6-8 |
|  | 13f | Describe any sensitivity analyses conducted to assess robustness of the synthesized results. | | Pages 6-8 |
| Reporting bias assessment | 14 | Describe any methods used to assess risk of bias due to missing results in a synthesis (arising from reporting biases). | | Page 7 |
| Certainty assessment | 15 | Describe any methods used to assess certainty (or confidence) in the body of evidence for an outcome. | | Page 7 |
| **RESULTS** | | | |  |
| Study selection | 16a | Describe the results of the search and selection process, from the number of records identified in the search to the number of studies included in the review, ideally using a flow diagram. | | Pages 8-9 and Figure 1 |
|  | 16b | Cite studies that might appear to meet the inclusion criteria, but which were excluded, and explain why they were excluded. | | Figure 1 |
| Study characteristics | 17 | Cite each included study and present its characteristics. | | Pages 8-9 and Table 1 |
| Risk of bias in studies | 18 | Present assessments of risk of bias for each included study. | | Pages 13-15 and Figure 8 |
| Results of individual studies | 19 | For all outcomes, present, for each study: (a) summary statistics for each group (where appropriate) and (b) an effect estimate and its precision (e.g. confidence/credible interval), ideally using structured tables or plots. | | Pages 9-11 |
| Results of syntheses | 20a | For each synthesis, briefly summarise the characteristics and risk of bias among contributing studies. | | Pages 9-15 |
|  | 20b | Present results of all statistical syntheses conducted. If meta-analysis was done, present for each the summary estimate and its precision (e.g. confidence/credible interval) and measures of statistical heterogeneity. If comparing groups, describe the direction of the effect. | | Pages 9-15 |
|  | 20c | Present results of all investigations of possible causes of heterogeneity among study results. | | Pages 9-15 |
|  | 20d | Present results of all sensitivity analyses conducted to assess the robustness of the synthesized results. | | Pages 9-15 and Supplementary Material |
| Reporting biases | 21 | Present assessments of risk of bias due to missing results (arising from reporting biases) for each synthesis assessed. | | Pages 14-15 |
| Certainty of evidence | 22 | Present assessments of certainty (or confidence) in the body of evidence for each outcome assessed. | | Table 2 |
| **DISCUSSION** | | | |  |
| Discussion | 23a | Provide a general interpretation of the results in the context of other evidence. | | Pages 15-18 |
|  | 23b | Discuss any limitations of the evidence included in the review. | | Pages 15-18 |
|  | 23c | Discuss any limitations of the review processes used. | | Pages 15-18 |
|  | 23d | Discuss implications of the results for practice, policy, and future research. | | Pages 15-18 |
| **OTHER INFORMATION** | | | |  |
| Registration and protocol | 24a | Provide registration information for the review, including register name and registration number, or state that the review was not registered. | | Page 5 |
|  | 24b | Indicate where the review protocol can be accessed, or state that a protocol was not prepared. | | Page 5 |
|  | 24c | Describe and explain any amendments to information provided at registration or in the protocol. | | - |
| Support | 25 | Describe sources of financial or non-financial support for the review, and the role  of the funders or sponsors in the review. | | Page 20 |
| Competing interests | 26 | Declare any competing interests of review authors. | | Page 20 |
| Availability of data, code and other materials | 27 | Report which of the following are publicly available and where they can be found: template data collection forms; data extracted from included studies; data used for all analyses; analytic code; any other materials used in the review. | | Page - |

**Supplementary Table S2. Prior distributions used for Bayesian analysis of perineal hernia**

| **Outcome / Scale** | **Prior (Primary)** | **Prior (Sensitivity 1)** | **Prior (Sensitivity 2)** | **Justification** |
| --- | --- | --- | --- | --- |
| Perineal hernia (log OR) | μ ~ Normal(0, 2²);  τ ~ Half Normal(0.5) | μ ~ Normal(0, 0.82²);  τ ~ Half Normal(0.25) | μ ~ Normal(0, 4²);  τ ~ Half Normal(1.0) | The primary μ prior was weakly informative and neutral, allowing a broad range of plausible treatment effects.  Sensitivity 1 used a more skeptical μ prior and a lower heterogeneity prior to test robustness under conservative assumptions.  Sensitivity 2 used a wider μ prior and a higher heterogeneity prior to evaluate stability under more diffuse assumptions. |

**Supplementary Table S3.** Summary of baseline demographic, oncologic, and operative characteristics of patients undergoing minimally invasive or open abdominoperineal resection/extralevator abdominoperineal excision included in the meta-analysis.

| **Characteristic** | **MIS** | **Open** |
| --- | --- | --- |
| Patient Characteristics |  |  |
| Patients, n (%) | 249 (32.6) | 514 (67.4) |
| Male sex, n (%) | 132/229 (57.6) | 310/480 (64.6) |
| Age, years | 61.75 ± 13.62 | 59.45 ± 13.73 |
| BMI, kg/m² | 24.50 ± 5.23 | 25.20 ± 4.93 |
| Diabetes, n (%) | 12/174 (6.9) | 47/331 (14.2) |
| Smoking, n (%) | 50/174 (28.7) | 82/331 (24.8) |
| Follow-up, months | 32.76 ± 34.18 | 33.56 ± 24.37 |
| Tumor Location |  |  |
| Rectal cancer, n (%) | 217/229 (94.8) | 440/480 (91.7) |
| Anal cancer, n (%) | 4/229 (1.7) | 10/480 (2.1) |
| No cancer, n (%) | 8/229 (3.5) | 30/480 (6.2) |
| Neoadjuvant Therapy |  |  |
| Radiotherapy, n (%) | 57/174 (32.8) | 133/331 (40.2) |
| Chemotherapy, n (%) | 48/174 (27.6) | 126/331 (38.1) |
| Chemoradiotherapy, n (%) | 36/104 (34.6) | 8/140 (5.7) |
| AJCC Stage |  |  |
| Stage 0, n (%) | 19/70 (27.1) | 61/191 (31.9) |
| Stage I, n (%) | 20/174 (11.5) | 39/331 (11.8) |
| Stage II, n (%) | 55/174 (31.6) | 80/331 (24.2) |
| Stage III, n (%) | 63/174 (36.2) | 116/331 (35.0) |
| Stage IV, n (%) | 15/174 (8.6) | 31/331 (9.4) |
| Perineal Reconstruction / Closure |  |  |
| Linear/primary suture, n (%) | 58/70 (82.9) | 160/191 (83.8) |
| Purse-string suture, n (%) | 8/70 (11.4) | 5/191 (2.6) |
| TRAM flap, n (%) | 1/70 (1.4) | 14/191 (7.3) |
| Gracilis flap, n (%) | 1/70 (1.4) | 7/191 (3.7) |
| Other flap, n (%) | 1/70 (1.4) | 4/191 (2.1) |
| Operative Characteristics |  |  |
| Combined pelvic resection, n (%) | 21/229 (9.2) | 71/480 (14.8) |

Footnote: Data are presented as n (%) or mean ± standard deviation. Percentages were calculated using the number of patients with available data for each variable; therefore, denominators vary owing to incomplete reporting across studies. Tumor location and combined pelvic resection data were available for 229 patients in the minimally invasive surgery (MIS) group and 480 patients in the open group. AJCC stage and perineal reconstruction variables were reported by a subset of studies only. MIS = minimally invasive surgery; BMI = body mass index; AJCC = American Joint Committee on Cancer; TRAM = transverse rectus abdominis myocutaneous flap.

**Supplementary Table S4. Bayesian sensitivity analyses for perineal hernia**

| **Model** | **Prior for μ** | **Prior for τ** | **Posterior OR (mean)** | **95% CrI** | **P(MIV increases perineal hernia risk)** |
| --- | --- | --- | --- | --- | --- |
| **Primary analysis** | Normal(0,2²) | Half-Normal(0.5) | 4.04 | 1.96–8.36 | 99.9% |
| **Sensitivity 1** | Normal(0,0.8²) | Half-Normal(0.25) | 3.41 | 1.86–6.27 | 100.0% |
| **Sensitivity 2** | Normal(0,4²) | Half-Normal(1.0) | 4.23 | 1.79–10.14 | 99.7% |

**Supplementary Table S5. Detailed justification for risk of bias assessment according to the ROBINS-I tool.**

| **Study** | **D1** | **D2** | **D3** | **D4** | **D5** | **D6** | **D7** | **Overall** |
| --- | --- | --- | --- | --- | --- | --- | --- | --- |
| Black 2021 ^19^ | Moderate – multivariable adjustment | Serious – retrospective participant selection | Low – intervention clearly defined | Moderate – no protocolized care | Low – multiple imputation | Moderate – no blinded assessment | Moderate – no protocol | Serious |
| Shen 2022 ^20^ | Serious – no propensity matching | Serious – retrospective participant selection | Low – intervention clearly defined | Moderate – no protocolized care | Moderate – loss to follow-up | Low – blinded radiological review | Moderate – no protocol | Serious |
| Sayers 2015 ^21^ | Serious – no adjustment | Serious – retrospective participant selection | Low – intervention clearly defined | Moderate – no protocolized care | Moderate – incomplete reporting | Moderate – no blinded assessment | Moderate – no protocol | Serious |
| Kent 2021 ^22^ | Serious – no adjustment | Serious – retrospective participant selection | Low – intervention clearly defined | Moderate – no protocolized care | Moderate – incomplete reporting | Moderate – no blinded assessment | Moderate – no protocol | Serious |

**Footnote:** D1 = Bias due to confounding; D2 = Bias in selection of participants; D3 = Bias in classification of interventions; D4 = Bias due to deviations from intended interventions; D5 = Bias due to missing data; D6 = Bias in measurement of outcomes; D7 = Bias in selection of the reported result.

**Supplementary Table S6. Summary of findings and certainty of evidence assessment according to the GRADE approach.**

| **Outcome** | **Studies** | **Design** | **Risk of bias** | **Inconsistency** | **Indirectness** | **Imprecision** | **Publication bias** | **Relative effect (95% CI)** | **Certainty** |
| --- | --- | --- | --- | --- | --- | --- | --- | --- | --- |
| Perineal hernia | 4 | Observational | Serious | Not serious (I² = 0%) | Not serious | Not serious | Not assessed | OR 4.13 [2.24,7.61] | Very Low (●○○○) |
| Operative time (min) | 2 | Observational | Serious | Not serious (I² = 0%) | Not serious | Very serious | Not assessed | MD -41.7 [-60.8,-22.5] | Very Low (●○○○) |
| Hospital stay (days) | 3 | Observational | Serious | Very serious (I² = 86%) | Not serious | Serious | Not assessed | MD -2.5 days [-5.4,0.4] | Very Low (●○○○) |
| Intraoperative blood loss (mL) | 3 | Observational | Serious | Very serious (I² = 87%) | Not serious | Very serious | Not assessed | MD -156.5 ml [-298.4,-14.5] | Very Low (●○○○) |
| Readmissions | 2 | Observational | Serious | Not serious (I² = 0%) | Not serious | Serious | Not assessed | OR 1.41 [0.82, 2.42] | Very Low (●○○○) |

**Footnote:** MD = mean difference; OR = odds ratio; CI = confidence interval; USD = United States dollars. Certainty was graded using GRADE. Observational evidence was downgraded for risk of bias, publication bias was not performed due to the small number of included studies limited formal assessment.

**Supplementary Figure S1.** Baujat plot demonstrating study-specific contribution to heterogeneity for hospital stay.

**Supplementary Figure S2.** Leave-one-out sensitivity analysis for hospital stay

**Supplementary Figure S3.** Baujat plot demonstrating study-specific contribution to heterogeneity for Intraoperative blood loss .

**Supplementary Figure S4.** Leave-one-out sensitivity analysis for Intraoperative blood loss .
